# High-Temperature Mechanical and Dynamical Properties of *γ*-(U,Zr) Alloys

**DOI:** 10.3390/ma16072623

**Published:** 2023-03-26

**Authors:** Jiang-Jiang Ma, Xue-Fen Han, Xiao-Xiao Cai, Ruizhi Qiu, Olle Eriksson, Ping Zhang, Bao-Tian Wang

**Affiliations:** 1 Institute of High Energy Physics, Chinese Academy of Sciences (CAS), Beijing 100049, China; 2 Spallation Neutron Source Science Center (SNSSC), Dongguan 523803, China; 3 School of Physics and Information Engineering, Shanxi Normal University, Taiyuan 030031, China; 4 University of Chinese Academy of Sciences (UCAS), Beijing 100049, China; 5 Science and Technology on Surface Physics and Chemistry Laboratory, Mianyang 621908, China; 6 Department of Physics and Astronomy, Materials Theory, Uppsala University, Box 516, SE-75120 Uppsala, Sweden; 7 School of Physics and Physical Engineering, Qufu Normal University, Qufu 273165, China; 8 Institute of Applied Physics and Computational Mathematics, Beijing 100088, China

**Keywords:** γ-(U,Zr), mechanical properties, thermodynamic stability, dynamical structural factor

## Abstract

High-temperature body-centered cubic (BCC) γ-U is effectively stablized by γ-(U,Zr) alloys that also make it feasible to use it as a nuclear fuel. However, relatively little research has focused on γ-(U,Zr) alloys due to their instability at room temperature. The effect of Zr composition on its mechanical properties is not clear yet. Herein, we perform molecular dynamics simulations to investigate the mechanical and dynamical stabilities of γ-(U,Zr) alloys under high temperatures, and we calculate the corresponding lattice constants, various elastic moduli, Vickers hardness, Debye temperature, and dynamical structure factor. The results showed that γ-U, β-Zr, and γ-(U,Zr) are all mechanically and dynamically stable at 1200 K, which is in good agreement with the previously reported high-temperature phase diagram of U-Zr alloys. We found that the alloying treatment on γ-U with Zr can effectively improve its mechanical strength and melting points, such as Vickers hardness and Debye temperature, making it more suitable for nuclear reactors. Furthermore, the Zr concentrations in γ-(U,Zr) alloys have an excellent effect on these properties. In addition, the dynamical structure factor reveals that γ-U shows different structural features after alloying with Zr. The present simulation data and insights could be significant for understanding the structures and properties of UZr alloy under high temperatures.

## 1. Introduction

As a metallic nuclear fuel, uranium zirconium (UZr) alloy is being considered as a candidate fuel for fast reactors due to its advantages of having high thermal conductivity and evolution under burn-up [1,2,3,4]. Generally speaking, the proposed advanced technology fuel (ATF) with higher thermal conductivity is conducive to increasing the rate of heat transfer and reducing stored energy in the core of a reactor. For use in light water reactors (LWRs), the materials of the ATF must not only adapt to extreme conditions but also remain mechanically and dynamically stable [5,6]. Therefore, investigating the mechanical and dynamical properties of UZr alloy at high temperatures has important significance for accelerating the engineering applications of new nuclear fuel technology.

Pure uranium exhibits three different crystal structures: the orthorhombic α-phase at low temperatures, the tetragonal β-phase in the temperature range of 940–1045 K, and the BCC γ-phase above 1045 K [7]. Among these phases, the γ-phase displays the best technical properties as a nuclear fuel, such as good dimensional stability, higher corrosion resistance, and isotropic expansion during irradiation [8,9]. Unfortunately, it is difficult for pure U to exist as γ-U in reactor operating and fuel preparation conditions. In order to stabilize the γ-phase and produce good irradiation behavior, alloying U with other cubic metals (such as Zr, Mo, and Nb) has been proposed. As one of the most promising binary alloys, UZr can further decrease the stable γ-phase temperature and increase the solidus/liquidus temperature, making it more suitable for metallic nuclear fuels [10]. The widely accepted phase diagram of UZr alloy [11,12,13] has been extensively studied theoretically along with experimental thermodynamic assessments [14,15,16]. After alloying with Zr, only one stoichiometric compound, δ-UZr${}_{2}$, mixes with α-U and α-Zr under low temperatures. When the temperature is increased, δ-UZr${}_{2}$ undergoes a phase transition to γ-(U,Zr), which is divided into two BCC solid solutions, ($\gamma_{1}$ and $\gamma_{2}$), at a low Zr concentration and can be further demixed into α-U + $\gamma_{2}$ in the temperature range of 886–966 K. The pure γ-U, γ-(U,Zr), and β-Zr belong to the high-temperature BCC phase and are stable in all concentrations at T = 1200 K.

BCC γ-(U,Zr) exhibits isotropic thermal expansion and isotropic thermal conductivity [13,17], making it a leading reactor choice. In general, understanding the stability and evaluating the mechanical and thermodynamic properties of γ-(U,Zr) from the theoretical studies is essential for its applications in nuclear reactors. Nowadays, the density functional perturbation theory (DFPT) and quasiharmonic approximation (QHA) have proved essential in drawing up ground-state phonon dispersion relations and physical properties. For example, several first-principle calculations have been performed in the literature to study the ground-state properties of UZr alloy [18,19,20,21,22,23,24]. However, DFT calculations are typically performed statically, implying that the calculations are mainly applied at 0 K. Our previous studies [25] also proved, via calculating elastic constants, that UZr alloy is mechanically unstable under ambient conditions. Thus, in the literature, very few properties of high-temperature phase γ-(U,Zr) have been calculated and analyzed.

In our present work, molecular dynamics (MD) simulations were performed to calculate the mechanical and dynamical stability of γ-(U,Zr) alloy at a high temperature of T = 1200 K. The corresponding structural features, various elastic moduli, Vickers hardness, Debye temperature, and dynamical structure factor were determined. The influence of Zr concentrations on the mechanical properties was investigated. The dynamical structure factors and thermal neutron scattering cross-sections related to the lattice vibration properties were reported.

## 2. Computational Methods

**Figure 1:**
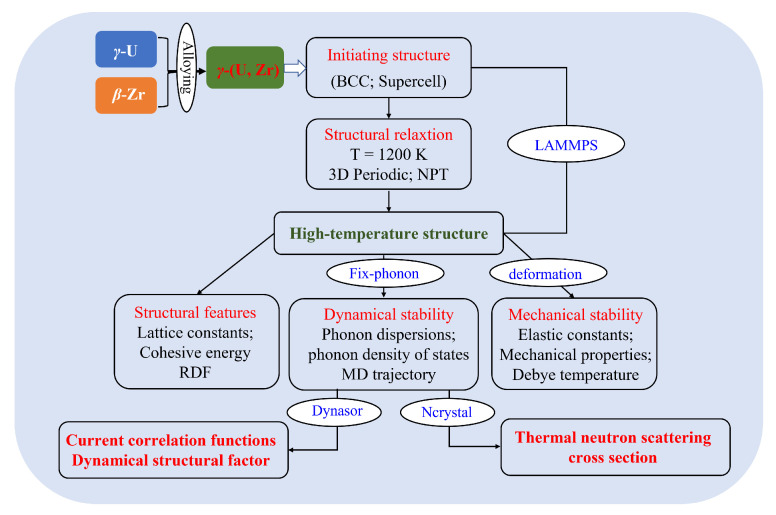
Internal workflow of this work. The blue text in the ellipse is the method or package used.

All MD simulations were carried out with LAMMPS [20], which is widely utilized in the field of molecular dynamics simulations to simulate diverse systems ranging from biological to solid-state physics, enabling simulations of particles at the atomic to the macroscopic level. The MEAM potential was used to describe the U-Zr systems [11]. Periodic boundary conditions were employed in all three directions in the simulation box. The systems were initially equilibrated under a temperature of 1200 K and at atmospheric pressure in an isotherm–isobaric ensemble, which lasted for 100 ps with a time step of 2 fs. After equilibration, we turned off the thermostat and barostat, and we recorded the velocities of all the atoms in the simulation. The workflow of this work is illustrated in Figure 1.

To analyze the thermodynamic stability of γ-phases at high temperatures, we calculated the phonon spectrum and phonon densities of states (PhDOSs) with the dynamical matrix. We adopted the fluctuation dissipation theory implemented in the Fix-Phonon package in the LAMMPS code [26]. The PhDOSs were calculated using a Monkhorst–Pack q-mesh of 80 × 80 × 80.

The dynamical structure factor simulations were performed using the Dynasor package from MD trajectories [27]. The dynamical structure factor, S(q,ω), can be estimated by the particle velocity correlation function, C(q,t). The current densities, j(r,t) and j(q,t), are defined as follows:

(1) $$j\left(r,t\right)=\sum_{i}^{N}v_{i}\left(t\right)\delta \left(r-r_{i}\left(t\right)\right),j\left(q,t\right)=\sum_{i}^{N}v_{i}\left(t\right)e^{iq\cdot r_{i}\left(t\right)},$$

where *N* is the number of particles, $v_{i}\left(t\right)$ and $r_{i}\left(t\right)$ are the velocity and position of particle *i* at time *t*, respectively. The current density can be decomposed into longitudinal and transverse parts, which can be expressed as $j_{L}\left(q,t\right)$ and $j_{T}\left(q,t\right)$:

(2) $$j_{L}\left(q,t\right)=\sum_{i}^{N}\left(v_{i}\left(t\right)\cdot \hat{q}\right)\hat{q}e^{iq\cdot r_{i}\left(t\right)},j_{T}\left(q,t\right)=\sum_{i}^{N}\left[v_{i}\left(t\right)-\left(v_{i}\left(t\right)\cdot \hat{q}\right)\hat{q}\right]e^{iq\cdot r_{i}\left(t\right)}.$$

where $\hat{q}$ denotes the unit vector. The current correlation functions, $C_{L}\left(q,t\right)$ and $C_{T}\left(q,t\right)$, can then be calculated by

(3) $$C_{L}\left(q,t\right)=\frac{1}{N}\langle j_{L}\left(q,t\right)\cdot j_{L}\left(-q,0\right)\rangle ,C_{T}\left(q,t\right)=\frac{1}{N}\langle j_{T}\left(q,t\right)\cdot j_{T}\left(-q,0\right)\rangle .$$

The current correlations can be transformed to the frequency domain via Fourier transformation. For the convenience of calculations, the scattering lengths are set to unity. The dynamical structural factor related to the frequency can be expressed as follows:

(4) $$S\left(q,\omega \right)=\frac{q^{2}C_{L}\left(q,\omega \right)}{\omega^{2}}.$$

## 3. Results

### 3.1. Crystal Structure

Pure γ-U and β-Zr are in the standard BCC space group of Im$\bar{3}$m, with one atom in the primitive cell. The space group of Im$\bar{3}$m has a bigger space group number (229th), which contains rich symmetry operations. The crystal can be formed by transferring the atoms and considering three-fold rotation and mirror symmetry. Relative to pure γ-U and β-Zr, the atoms in γ-(U,Zr) tend to be located randomly on the crystalline lattice, which breaks the crystal symmetry. This random atomic arrangement of different elements, also called chemical disorder, significantly alters the physical properties of materials [28,29,30]. In order to calculate the lattice constants at different Zr concentrations, we constructed a 24a × 24a × 24a supercell of a BCC structure for γ-(U,Zr), in which the atoms were randomly selected and replaced until the desired composition was achieved. During MD simulations, the structures initially underwent the minimum energy configurations and were heated up to 1200 K under the *NPT* ensemble. After relaxation, we calculated the cohesive energy, an essential parameter for determining the stability of solids. For different Zr concentrations, we found that the crystal structures still maintained the BCC structure.

**Figure 2:**
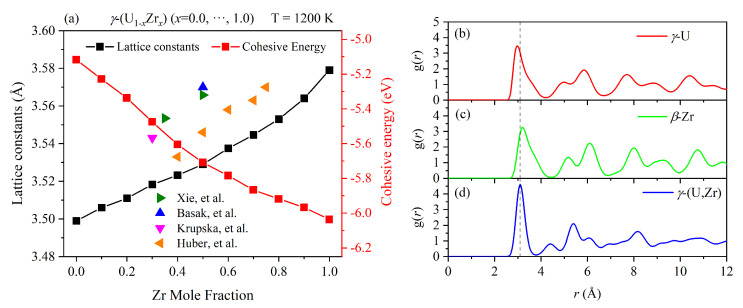
(**a**) The calculated lattice constant and cohesive energy versus Zr mole fraction for γ-(U,Zr) alloys by MD, the experimental lattice parameters of γ-(U,Zr) from previous studies [31,32,33,34] are also plotted, for comparison; the calculated radial distribution function for (**b**) γ-U, (**c**) β-Zr, and (**d**) γ-(U,Zr) at a high temperature of 1200 K.

The dependence of lattice constants and the cohesive energy of γ-(U${}_{1-x}$Zr${}_{x}$) on the Zr concentrations are displayed in Figure 2a. For comparison, we also plotted the lattice parameter of γ-(U,Zr) in previous experimental studies [31,32,33,34]. The lattice constant almost linearly increased, while the cohesive energy linearly decreased, when the Zr mole fraction increased. The variation of the lattice constant with increased Zr concentrations was in good agreement with experimental trends. Moreover, our estimated concentration dependence of the lattice parameters agreed well with Vegard’s law and evolved according to the difference in the bond length and the ion size between the U and Zr ions.

To understand the structural features of γ-U, β-Zr, and γ-(U,Zr) at high temperatures, we calculated their radial distribution functions (RDFs) and the results are presented in Figure 2b–d. Due to the crystal structure and symmetry being the same, the shape of the curve of γ-U is almost the same as that of β-Zr. However, the RDF of γ-(U,Zr) has very distinct peaks. In particular, the nearest-neighbor peak position of the RDF of γ-(U,Zr) is located in an intermediate location between those of γ-U and β-Zr, as shown by the dotted line. This indicated the orders of the nearest-neighbor distances: $R_{\mathrm{Zr}-\mathrm{Zr}}>R_{U-\mathrm{Zr}}>R_{U-U}$, at least for small bond distances. The broadness of the RDF peaks is an indication of thermal effects. Since the first peak of γ-(U,Zr) is more sharp than those of γ-U and β-Zr, we concluded that the atoms in γ-(U,Zr) had smaller displacements at their mean positions. This indicated that U-Zr alloying could potentially improve the structural stability of the BCC phase.

### 3.2. Mechanical Stability and Properties

In order to verify the effectiveness of γ-U alloying with Zr, we calculated the elastic properties to verify the mechanical stability of γ-(U,Zr) at 1200 K. Due to the cubic symmetry of γ-(U,Zr), there were three independent elastic constants $C_{ij}$, which are $C_{11}$, $C_{22}$, and $C_{44}$. The deformation method was used in this work. The system was equilibrated in the NPT ensemble at 1200 K. After obtaining the high-temperature structure, a series of simulations were performed with deformation to calculate the equilibrium stress tensor. The $C_{ij}$ elements could then be extracted from Hooke’s law $\sigma_{i}=C_{ij}\epsilon_{j}$, where σ and ϵ are strain and stress tensors, respectively. Our calculated elastic constants are presented in Table 1. For comparison, the previous results of γ-U [35,36] and our reported values of UZr from DFT at 0 K [25] are also listed. As shown, our predicted results of γ-U from MD simulations were close to the previous experimental values [35], which were measured for polycrystalline γ-phase U-8wt% Mo. This shows that this method is reliable for evaluating the mechanical properties of high-temperature phases.

According to the mechanical stability criteria for the cubic crystal system as in [37]

(5) $$C_{11}>\left|C_{12}\right|,C_{11}+2C_{12}>0,C_{44}>0.$$

The high-temperature elastic constants of γ-(U,Zr) satisfy such criteria, whereas the results of γ-UZr [25] and γ-U [36] at 0 K did not meet these criteria. Thus, the γ-(U,Zr) are mechanically stable at high temperatures.

Moreover, the elastic constants afford information on the mechanical strength and hardness. Armed with these outcomes, we further calculated the various moduli, Vicker’s hardness, elastic wave velocities, and Debye temperatures for γ-(U${}_{1-x}$Zr${}_{x}$) (x=0,0.25,0.5,0.75) at 1200 K and present them in Table 1. The bulk modulus B and shear modulus G can be qualitatively assessed from the Voigt–Reuss–Hill (VRH) approximations [38,39,40]:

(6) $$B=\frac{C_{11}+2C_{12}}{3},G=\frac{G_{v}+G_{R}}{2},$$

where $G_{v}$ and $G_{R}$ represent the Voigt and Reuss forms of the Shear modulus, respectively. The $G_{v}$ and $G_{R}$ of cubic crystals are obtained from the elastic constants:

(7) $$G_{v}=\frac{C_{11}-C_{12}+3C_{44}}{5},G_{R}=\frac{5\left(C_{11}-C_{12}\right)C_{44}}{4C_{44}+3\left(C_{11}-C_{12}\right)}.$$

The Young’s modulus E is used to describe the stiffness of materials and is calculated using the following equation:

(8) $$E=\frac{9\mathrm{BG}}{3B+G}.$$

It can be seen that the B, G, and E of γ-U can be enhanced after alloying with Zr. This means that there was an increase in the mechanical strength, obviously. Hardness is also one of the indicators to measure the characterization of solids. The Vickers hardness ($H_{v}$) can be predicated from the following equation [41]:

(9) $$H_{v}=0.92{\left(G/B\right)}^{1.137}G^{0.708}.$$

Our results for the Vickers hardness, $H_{v}$, for γ-(U,Zr) alloys were greater than those of γ-U under high temperature conditions. This means that the hardness of γ-U can be greatly enhanced by alloying with Zr. In addition, the Debye temperature $\Theta_{D}$ is a significant thermodynamic parameter related to the elastic modulus and melting point within the Debye theory; it can be obtained from the averaged sound velocity [42,43]:

(10) $$\Theta_{D}=\frac{h}{k_{B}}{\left(\frac{3n}{4\pi \Omega }\right)}^{1/3}v_{m}.$$

Approximately, $v_{m}$ can be given by [44]:

(11) $$v_{m}={\left[\frac{1}{3}\left(\frac{2}{v_{t}^{3}}+\frac{1}{v_{l}^{3}}\right)\right]}^{-1/3},$$

where $v_{t}=\sqrt{G/\rho }$ (ρ is the density) and $v_{l}=\sqrt{(3B+4G)/3\rho }$. Compared to pure γ-U, the $\Theta_{D}$ of γ-(U,Zr) can be effectively increased. The larger $\Theta_{D}$ of γ-(U,Zr) means that the melting point of γ-U was improved after alloying with Zr. An increased melting point is beneficial to the application of metallic U as a nuclear fuel.

The mechanical properties of an alloy are also manipulated by the concentrations. The mechanical properties of γ-(U${}_{1-x}$Zr${}_{x}$) (x=0.25,0.5,0.75) alloys were derived, as shown in Table 1. There were evident Zr concentration-dependent behaviors in those mechanical properties. For example, the $H_{v}$ and $\Theta_{D}$ increased with an increase in Zr concentration, which further verified the significant improvement of Zr in terms of the mechanical properties and melting points of γ-(U,Zr) alloys.

### 3.3. Dynamical Stability and Properties

**Figure 3:**
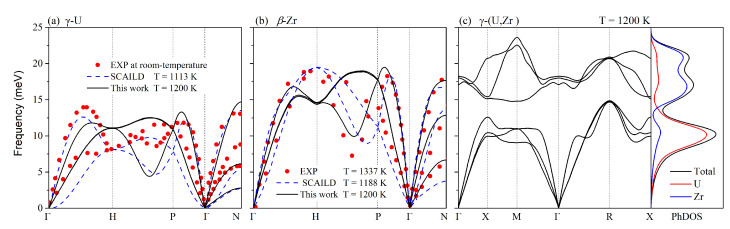
Phonon dispersions for (**a**) γ-U, (**b**) β-Zr, and (**c**) γ-(U,Zr) at 1200 K. Experimental (the red dots) and other theoretical (the blue dotted line) results for γ-U and β-Zr, the PhDOSs for γ-(U,Zr) are also plotted. The experimental and theoretical values of γ-U are from Refs. [45,47], respectively. In addition, the experimental and theoretical values of β-Zr are from Refs. [46,48], respectively.

In order to study the dynamical stability, we also calculated the phonon dispersions at T = 1200 K. The initial system was firstly equilibrated at 1200 K with zero external pressure in an NPT ensemble before calculating the dynamical properties. In Figure 3a,b, we compare the phonon dispersion curves at 1200 K for γ-U and β-Zr obtained by MD simulations to the experimental data mentioned above [45,46] and the theoretical high-temperature phonon spectra derived using the SCAILD method [47,48]. Due to our theoretical calculations being performed at elevated temperatures, some differences should exist when comparing them to the experimental data. Apart from that, our calculated curves were in good agreement with the experimental results. The dispersion curves showed a large softening along the H–P direction and the vibrational mode showed linear curvature near the zone center, which agreed well with the experimental results. Compared to β-Zr, γ-U has lower phonon modes and phonon group velocities, which can be attributed to its lower Debye temperature, stemming from its larger atomic mass.

In previous studies [47,49,50], the phonon dispersions of γ-U and β-Zr exhibited several imaginary phonon modes, showing instability in the BCC phase under low temperatures. One recent study by Qian et al. [50] has showed that the potential-energy surface of β-Zr has a double-well shape and the equilibrium state of the BCC structure is at a saddle point. Under high temperatures, the equilibrium position is the dynamical average between the two local minima. Thus, temperature can stabilize the BCC phase. Heiming et al. [46] measured the phonon dispersion of β-Zr at several high temperatures using the thermal neutron three-axis spectrometer, which indicated the dynamical stability of β-Zr at high-temperatures.

Subsequently, we calculated the phonon dispersion curves and phonon density of states (PhDOSs) for γ-(U,Zr) at 1200 K, as shown in Figure 3c. Since there are two atoms in its primitive cell, the γ-(U,Zr) has six phonon branches. As shown, the highest vibrational frequency of γ-(U,Zr) was larger than that of γ-U, indicating a stronger interatomic interaction in γ-(U,Zr). By alloying with Zr, the phonon frequency of U was increased, as indicated by the PhDOSs. There was no evident acoustic–optical coupling, due to the existence of a continuous phonon band gap between its optic and acoustic modes. In addition, all the phonon frequencies of these BCC phases at high temperatures were positive. The crystal structures were still able to maintain the stable BCC phases after the structural relaxation at T = 1200 K, which suggests dynamic stability under high temperatures.

As mentioned above, the vibration of atoms can provide critical insights for understanding the structural and thermodynamic properties of materials. In general, the atomic vibrations can be probed using inelastic neutron scattering experiments due to the energy and momentum of thermal/cold neutrons being comparable to those of phonons. Inelastic neutron scattering has been widely used to verify the correct structure and vibrational dynamics [51]. The thermal neutron scattering law and dynamic structure factor are excellent ways to capture the effects of neutron cross-section. In order to investigate the dynamical properties of the BCC phase under high temperatures, we calculated the dynamical structure factors S(q,ω) of γ-U, β-Zr and γ-(U,Zr) along their possible directions.

**Figure 4:**
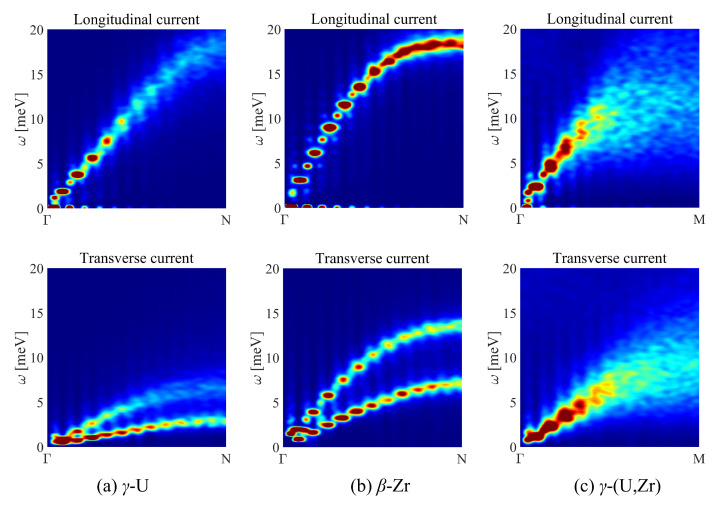
Longitudinal current and transverse current for (**a**) γ-U, (**b**) β-Zr, and (**c**) γ-(U,Zr) as a function of ω at a high temperature of 1200 K.

The calculated contour maps of the longitudinal and transverse currents at 1200 K for γ-U, β-Zr, and γ-(U,Zr) are plotted in Figure 4. Compared with the results of their phonon spectra, the current dispersion relations clearly showed the longitudinal acoustic (LA) and TA branches for γ-U and β-Zr at high temperatures. However, for γ-(U,Zr) alloy, the dispersion relation was not that evident. There was only one mode in its transverse current along the Γ-M direction, suggesting a superposition of adjacent vibration in intensity, rather than a degeneracy. Compared with γ-U and β-Zr, such a dispersion mode indicated stronger acoustic phonon scattering between the vibrations of the atoms in γ-(U,Zr) alloy.

**Figure 5:**
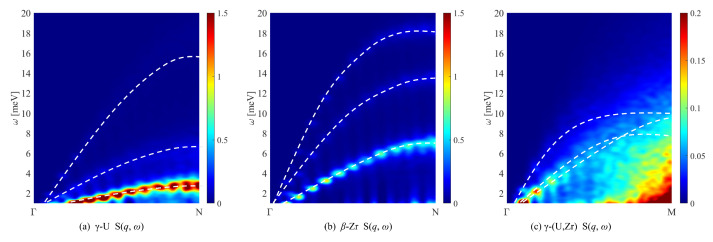
The dynamical structure factor for (**a**) γ-U, (**b**) β-Zr, and (**c**) γ-(U,Zr) as a function of ω at a high temperature of 1200 K. The white dashed lines correspond to the phonon dispersion relations.

We calculated the contour maps of the dynamic structure factor on the plane of (ω, *k*) for γ-U, β-Zr, and γ-(U,Zr) at 1200 K and present the results in Figure 5. Clearly, the dynamic structure factors S(q,ω) were consistent with the phonon dispersion relations. The intensity of S(q,ω) was stronger at smaller ω, which was caused by the factor of 1/ω in the cross-section. The cross-section here mainly refers to coherent single phonon scattering, and the expression can be found in Ref. [52]. It should be noted that the spectral signatures of the dynamical structure factor presented a discontinuity distribution due to the limitations of model size in the simulation. As shown in Figure 5a,b, the pure metallic γ-U and β-Zr had similar dynamic characters because their crystal structures were the same. However, the dispersion of γ-(U,Zr) was evidently different with the pure metal, which indicated the unique structure feature of UZr alloy comparing with γ-U and β-Zr.

Moreover, the dynamical structure factor and the thermal neutron scattering cross-sections can also reflect the structural and dynamical properties at the atomic level. Understanding the scattering behavior is of critical importance for its application to nuclear reactors. Here, we simulated the neutron coherent elastic, incoherent, and inelastic scattering of γ-(U,Zr) using Ncrystal [53], which is an open source software for the calculation of thermal neutron transport based on the parameters of a crystal unit cell.

According to Born approximation and Fermi potential, the differential cross-sections for thermal neutron scattering can be deduced by

(12) $$\frac{d^{2}\sigma_{{\vec{k}}_{i}\Rightarrow {\vec{k}}_{f}}}{d\Omega_{f}dE_{f}}=\frac{k_{f}}{k_{i}}S\left(q,\omega \right),$$

(13) $$S\left(q,\omega \right)=\frac{1}{2\pi \hbar }\sum_{j,j^{\prime }=1}^{N}b_{j}b_{j^{\prime }}\int_{-\infty }^{\infty }dt\langle e^{-iq\cdot R_{j^{\prime }}\left(0\right)}e^{iq\cdot R_{j}\left(t\right)}\rangle e^{-i\omega t}.$$

where q is the neutron wavevector and ω is the neutron frequency. The equations $q=k_{i}-k_{f}$ and $\hbar \omega =E_{f}-E_{i}$ stand for the total momentum and energy transfer between a neutron and scattering atoms, respectively. The subscripts *i* and *f* stand for the incident and final states of a neutron, respectively, and *j* and $j^{\prime }$ stand for the indexes of the scattering atoms. The scattering function S(q,ω), defined in Equation (13), is the integration over the correlation between the position of the nucleus *j* at time *t* and the position of the nucleus $j^{\prime }$ at time 0 with respect to *t* and the summary of the nuclei *j* and $j^{\prime }$. The neutron scattering length of the *j*^th^ nucleus $b_{j}$ is an effective parameter to describe the neutron–nucleus interaction. When ${\vec{k}}_{i}={\vec{k}}_{f}$, the elastic scattering happens and there is no energy or phonon exchanges between the neutron and scattering target atoms. When ${\vec{k}}_{i}\neq {\vec{k}}_{f}$, the inelastic scattering happens, which is the emission or absorption of phonons. The total scattering function can be expressed as follows:

(14) $$S\left(q,\omega \right)=S_{\mathrm{coh}}\left(q,\omega \right)+S_{\mathrm{inc}}\left(q,\omega \right),$$

where $S_{\mathrm{coh}}(q,\omega$) refers to the coherent scattering, which is an interference effect depending on the correlation between the positions of the same nucleus at different times (self correlation, sum over $j=j^{\prime }$) as well as between the positions of different nuclei at different times (distinct correlation, sum over $j\neq j^{\prime }$). The function $S_{\mathrm{inc}}\left(q,\omega \right)$ represents the incoherent scattering, which depends only on self correlation, thereby giving no interference effects [52]. NCrystal calculates relevant reflection planes and structure factors, then chooses the model according to the configuration. The inelastic cross-section is implemented through scattering kernels according to incoherent approximation, and an example of it along with its method are given in Refs. [53,54].

**Figure 6:**
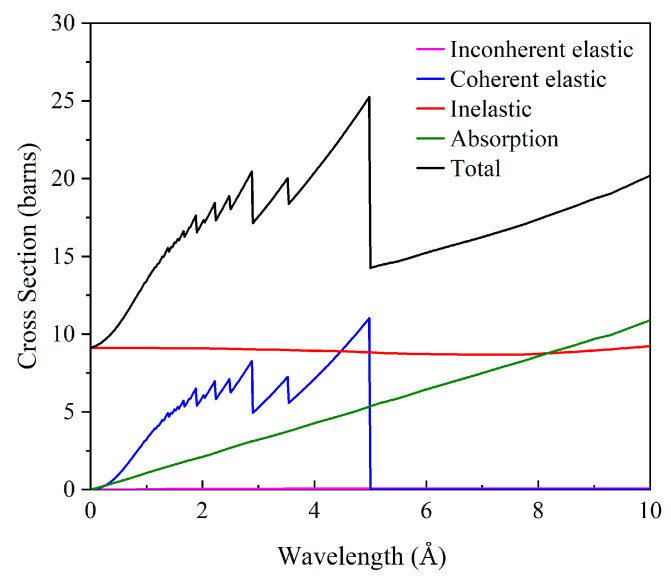
Thermal neutron scattering cross-section and scattering angle for γ-(U,Zr) alloy generated from the PhDOSs calculated using the MEAM method, at T = 1200 K.

The results of coherent elastic, incoherent elastic, inelastic, absorption, and total cross-section of γ-(U,Zr) at 1200 K through the vibrational density of states (vdos) model are presented in Figure 6. It is clear that the obvious Bragg edges and peaks under 5 Å stemmed from Bragg diffraction. This satisfied the Bragg condition when neutrons scattered from planes of crystal. The Bragg cutoff was shown in the coherent elastic cross-section beyond 5 Å, while the incoherent elastic scattering was negligible in the given range. The inelastic cross-section was almost constant as the wavelength increased at the beginning and inclined slowly after 8 Å. In the low-wavelength region, the cross-sections were dominated by neutron coherent elastic and inelastic scattering, whereas only the latter existed beyond 5 Å. It should be noted that the resonance cross-section, which is related to nuclear structure, was not considered in this paper.

## 4. Conclusions

In summary, we have systematically performed MD simulations on the mechanical and dynamical properties of γ-(U,Zr) alloys at a temperature of 1200 K. The results indicated that γ-U, β-Zr, and γ-(U,Zr) were mechanically and dynamically stable at 1200 K. For each different Zr concentration, we found that the crystal structures still maintained the BCC structure at 1200 K, and the lattice constants increased linearly with an increasing Zr mole fraction. Through the calculation of elastic constants, various moduli, Poisson’s ratio, hardness, and Debye temperatures, we proved that the mechanical strength and melting point of γ-U can be significantly improved after alloying with Zr. The stoichiometry of γ-(U${}_{1-x}$Zr${}_{x}$) alloy had a significant influence on the mechanical properties at high temperatures. Based upon these data, we further obtained the dynamical structure factor and thermal neutron scattering cross-sections of γ-(U,Zr) alloy at high temperatures.

These theoretical results of mechanical and dynamical properties provide a reference for the design and application of UZr as a nuclear fuel. We hope our work can provide some directions and information for scientists in the fields of nuclear reactors and neutron scattering. Due to approximations in potential, limitations in simulation size, and statistical uncertainty of using the MD method, these results still need to be verified in future experimental measurements, such as by using inelastic neutron scattering and a powder diffractometer.

## Figures and Tables

**Table 1:** Calculated elastic constants (GPa), bulk modulus B (GPa), shear modulus G (GPa), Young’s modulus E (GPa), Vickers hardness $H_{v}$ (GPa), transverse $v_{t}$ (m/s), longitudinal $v_{l}$ (m/s), average $v_{m}$ (m/s) sound velocities, and Debye temperature $\Theta_{D}$(K) for γ-(U${}_{1-x}$Zr${}_{x}$) (x=0,0.25,0.5,0.75) at a temperature of 1200 K, respectively. For γ-U, the elastic constant error (%) and polycrystalline γ-phase U-8wt% Mo [35] comparisons are also listed.

	γ-U (%)	γ-(U${}_{0.75}$Zr${}_{0.25}$)	γ-(U${}_{0.5}$Zr${}_{0.5}$)	γ-(U${}_{0.25}$Zr${}_{0.75}$)	γ-U [35]	γ-U [36]	γ-UZr [25]
$C_{11}$	112.5 (18.2%)	144.5	157.4	176.4	137.6	86	57.5
$C_{12}$	77.2 (7.4%)	76.2	68.5	44.8	83.4	155	78.2
$C_{44}$	34.4 (53.2%)	26.9	59.3	69.1	16.2	37	64.7
B	88.9	98.9	98.1	88.7			77.8
G	26.3	50.1	52.8	67.7			
E	71.9	128.5	134.4	162.0			
$H_{v}$	2.338	6.769	7.549	13.407			
$v_{t}$	1195.4	1801.5	2054.8	2667.3			
$v_{l}$	2594.2	3277.5	3670.4	4335.3			
$v_{m}$	1346.8	2008.1	2287.2	2943.2			
$\Theta_{D}$	144.3	214.4	243.6	311.3			

The results from [25] were calculated using the DFT method. The results from [35] were measured from polycrystalline γ-phase U-8wt% Mo using the resonant ultrasound spectroscopy (RUS) technique. The results of [36] were calculated using the DFT method.

## Data Availability

The data presented in this study are available on request from the corresponding authors. The data are not publicly available due to ongoing research in the project.
